# Cesarian section and long-term outcomes for cownose rays (*Rhinoptera bonasus*)

**DOI:** 10.3389/fvets.2024.1411769

**Published:** 2024-09-04

**Authors:** Chris Buckner, Robert H. George, Frank Bulman, Jared Durrett, Tim Handsel, Jennifer T. Wyffels

**Affiliations:** ^1^Ripley’s Aquarium of the Smokies, Gatlinburg, TN, United States; ^2^Ripley’s Aquarium of Myrtle Beach, Myrtle Beach, SC, United States; ^3^Ripley’s Aquariums, Orlando, FL, United States

**Keywords:** dystocia, reproduction, elasmobranch, parturition, histotroph

## Abstract

Cownose rays (*Rhinoptera bonasus*) are schooling rays commonly displayed in large groups in public aquariums. They are long-lived, have an annual reproductive cycle, and readily breed in managed care with most pregnancies culminating with the unaided and successful birth of a single neonate. Occasionally, females are observed to have prolonged pregnancies or suffer dystocia during parturition and intervention via a cesarian section (C-section) is required to deliver the neonate. Monthly reproductive monitoring at Ripley’s Aquarium of the Smokies using ultrasound to stage pregnancies allows for the prediction of anticipated due dates and guides the decision to assist with delivery. Recognizing when to assist birth and best practices for performing C-section are important for the reproductive health, sustainability, and longevity of this species in managed care. This report describes a surgical technique for C-section in cownose rays and includes short-term complications and long-term outcomes for females.

## Introduction

Cownose rays (*Rhinoptera bonasus*) have been part of the elasmobranch assemblage at Ripley’s Aquarium of the Smokies since its opening in December 2000. Male and female cownose rays are maintained together in an 86,000-gallon exhibit that includes a shallow 2-ft-deep touch pool to facilitate guest interaction with the rays through enrichment programs, connected to a 20-ft-deep pool. The rays are housed with southern stingrays (*Hypanus americanus*), spotted eagle rays (*Aetobatis narinari*), bonnethead sharks (*Sphyrna tiburo*), whitespotted bamboo sharks (*Chiloscyllium plagiosum*), and epaulette sharks (*Hemiscyllium ocellatum*).

Cownose rays are long-lived and reproduce annually. Females have a functional left ovary and uterus (1–3) and, except in rare cases (4), give birth to a single neonate after a nearly year-long gestation (1–3). Collectively, this combination of life history traits makes cownose rays particularly susceptible to exploitation from fishing pressure and habitat loss or degradation (5). Maintaining healthy and self-sustaining populations of cownose rays in managed care is a goal of the aquarium that also supports the conservation of this species. The cownose rays in Ripley’s Aquarium of the Smokies breed readily, and their reproductive status has been monitored monthly via ultrasonography since 2005. Females are assigned a reproductive stage (stages 0–5) based on the contents and appearance of the uterus as has been previously described: (0) uterus is not visualized indicating an immature ray; (1) uterus with or without fluid and without an embryo or solid material; (2) uterus with undifferentiated material present in the fluid in the uterus and without organized motion; (3) differentiated embryo floating in uterine fluid with or without organized motion; (4) differentiated embryo present in the uterine fluid, embryo larger than the ultrasound field of view and organized motion detected including the movement of the gill arches indicating respiration and heart valve; and (5) large fetus with hyperechoic folded wings causing displacement and misshaping of the spiral valve (6). When females reach stage 5, late-term pregnancy, they are transferred from the main exhibit to a 20-ft diameter round holding pool for parturition. Occasionally, females stagnate in stage 5 and develop dystocia, requiring surgical intervention to deliver the young by cesarian section (C-section). This procedure is lifesaving for the female and when the timing is correct, also for the neonate. A review of the past 20 years of cownose ray reproduction has allowed for the development and refinement of a C-section procedure for the species and tracking of long-term reproductive outcomes for female cownose rays (*n* = 7) that underwent C-sections.

## Materials and methods

### Animals

A retrospective review of husbandry and medical records extracted the number of pregnancies and C-sections for resident cownose rays from 2005–2024 at Ripley’s Aquarium of the Smokies. Clinical signs of dystocia, stage of pregnancy (0–5), surgical procedure, and postoperative and long-term outcome information up to 20 years were noted for females requiring intervention.

### Case selection

A normal pregnancy culminates with reproductive stage 5, with the fetus filling the uterus, which in late pregnancy extends across the ray’s midline, causing displacement and crowding of the spiral intestine (6). Females are are normally observed in stage 5 for 1–2 months at the end of their 11–12 months of gestation (6). The parturition date for the previous pregnancy—in combination with exposure to males postpartum—was used to estimate the subsequent parturition date because females were often mated soon after giving birth. Females that persisted longer than 2 months in stage 5 or that had surpassed their expected parturition date were considered at risk of dystocia. The normal position of the fetus within the uterus was a caudal presentation, with the wings folded dorsally and the tail folded in a cranial direction over the body. Incorrect orientation of the fetus may contribute to clinical signs of dystocia.

**Figure 1:**
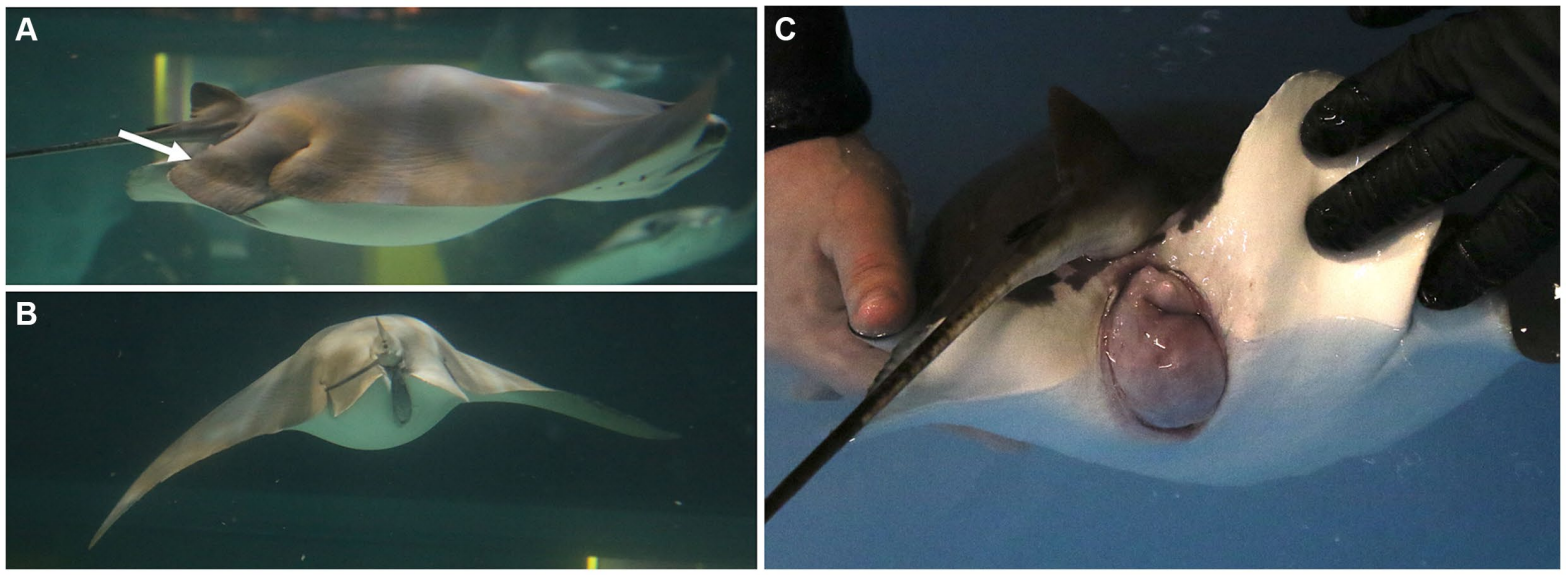
Females may exhibit external signs of dystocia characterized by **(A)** pelvic fin edema (arrow), **(B)** marked coelomic distention, and/or **(C)** cloacal protrusion, edema, and/or bruising. These signs are helpful for deciding the timing to intervene and deliver young by C-section.

Pregnant cownose rays were examined monthly via ultrasonography to confirm that their young were viable and to monitor their size and orientation relative to the female’s coelom. The decision to perform a C-section was based on both maternal and fetal factors. Externally, females may have exhibited external signs of dystocia characterized by marked coelomic distention, pelvic fin edema, and cloacal protrusion, edema, and/or bruising (Figure 1). Ultrasound of the dam was used to evaluate the fetus for a heartbeat and the presence or absence of movement of the gill arches, indicating respiration and/or fetal movement.

### Surgical procedure

A pregnant ray requiring intervention is removed from her exhibit using dip nets and placed in a holding tank containing oxygenated saltwater from her exhibit. A second anesthetic tank with water from the same source is then treated with tricaine methanesulphonate (MS-222) (75–100 ppm, Syndel, Ferndale, Washington, United States) buffered 4:1 with sodium bicarbonate. Pure oxygen is delivered to the anesthetic tank via an airstone. Once the MS-222 and bicarbonate have dissolved, the ray is added. The anesthetic plane is deemed sufficient when the ray is minimally responsive to touch while maintaining spiracular respiration, the intake of water into the dorsal spiracles, which flows over the gill arches and out of the gill slits ventrally.

**Figure 2:**
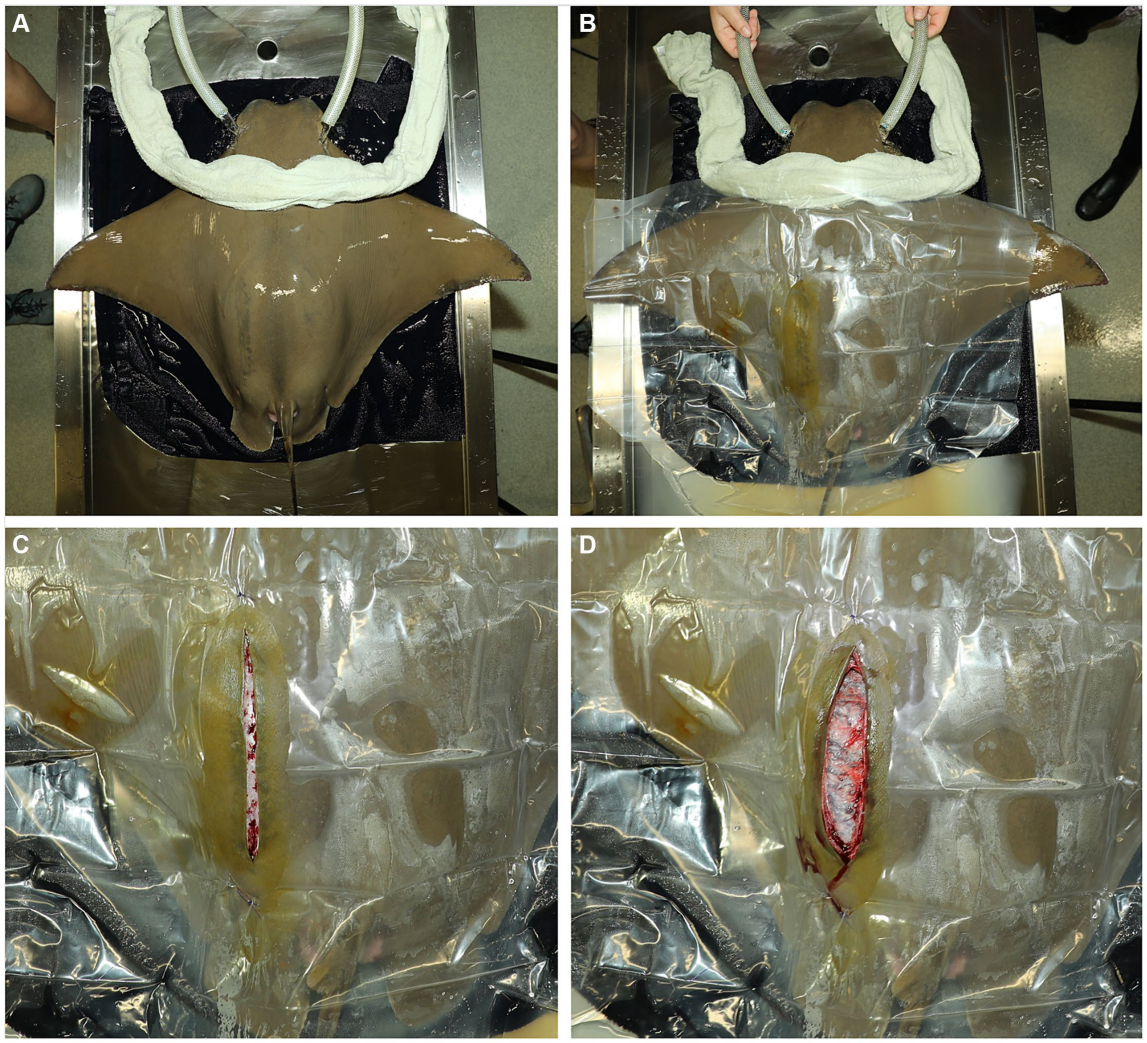
**(A)** An anesthetized ray in ventral recumbency on premoistened towels covering the surgical surface of a custom and portable stingray medical treatment cart. Anesthetic water is pumped through the ray’s spiracles. A premoistened cotton towel is laid caudal to the spiracles to block potential overflow anesthetic water from contaminating the surgical site. **(B)** The surgery site is prepped with dilute iodine. The ray is covered with a sterilized plastic drape that is sutured to the skin with a single, simple interrupted suture at the cranial and caudal end of the fenestration. **(C)** A left paramedian incision is made into the dorsal coelom in the left paralumbar fossa over the uterus using a number 10 blade. **(D)** The uterus is directly under the body wall.

The ray is lifted out of the anesthetic tank and placed in ventral recumbency on premoistened towels covering the surgical surface of a custom and portable stingray medical treatment cart adjacent to the holding tank (Figure 2A). A submersible pump placed in the anesthetic tank pumps water through a ¾-inch polypropylene hose to a T adapter from which ¾-inch hoses are placed into the ray’s spiracles. A ball valve allows for adjustment of the flow rate. Anesthetic water flows through the spiracles, cascades over the gills, and drains from the ray’s gill slits as well as her mouth. The anesthetic water drains from the top tray of the medical cart into the anesthetic tank on the bottom tray of the cart and is recycled back into the anesthetic holding tank. Anesthetic concentration during the procedure is maintained at 75 ppm MS-222. The ray’s respiratory rate is monitored throughout the procedure. If the rate decreases 25% from the baseline determined before respiratory rate recover, fresh water is added gradually to reduce the MS-222 concentration until the anesthetic-free seawater.

**Figure 3:**
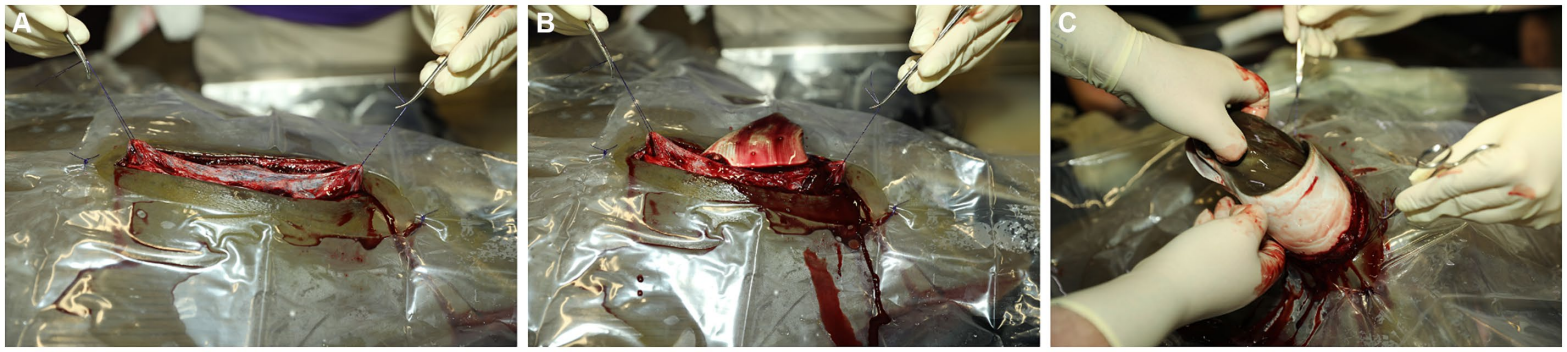
**(A)** Stay sutures are placed at the cranial and caudal terminus of the uterus using 3–0 PDS. Using the sutures and applying gentle traction, the uterus is partially exteriorized. **(B)** A stab incision is made into the uterus and extended with scissors. The neonate is quickly identified and removed from the uterus manually using gentle traction on a wing to rotate the fetus to be oriented as needed to **(C)** grasp the dorsal rim of the spiracle openings.

A premoistened cotton towel is laid caudal to the spiracles to block potential overflow anesthetic water from contaminating the surgical site. The left paralumbar area is sanitized using gauze soaked in povidone-iodine surgical prep solution and applied using light pressure starting at the incision site and circling outward. The povidine-iodine application is repeated twice more, and a sterilized plastic drape is sutured to the skin with a single, simple interrupted suture at the cranial and caudal end of the fenestration (Figure 2B). A left paramedian incision, 6–10 inches depending on the size of the neonate, through the skin, musculature, and peritoneum is made into the dorsal coelom in the left paralumbar fossa, over the uterus using a number 10 blade (Figure 2C). Care should be taken when extending the incision caudally, as the kidneys are in the caudodorsal portion of the coelom. Ultrasonography can be used to locate the cranial tip of the kidneys for reference. The uterus is directly under the body wall in this area (Figure 2D), and stay sutures are placed at the cranial and caudal ends using a 3–0 polydioxanone suture (PDS). Using the sutures and applying gentle traction, the uterus is partially exteriorized (Figure 3A). This position minimizes the spillage of uterine contents into the coelom.

A stab incision is made into the uterus using a number 10 blade, and the incision is extended with Mayo scissors to a length that will allow extraction of the neonate. The neonate is grasped gently by a wing (Figure 3B), rotated to be oriented head first, and extracted manually using gentle traction on the dorsal rim of the spiracle openings (Figure 3C). The visible uterine blood vessels that may be severed when making the incision do not require ligation, thanks to uterine wall contracture.

**Figure 4:**
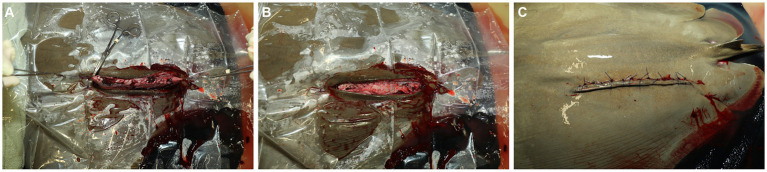
**(A)** The uterus is partially exteriorized using the stay sutures, and residual uterine fluid (histoptroph) is removed in preparation for closing the incision. **(B)** The uterus is closed, and the stay sutures are removed. **(C)** The skin is closed with 2–0 PDS on a cutting needle in a horizontal mattress pattern.

The uterus is held up and slightly out of the body wall opening using the stay sutures, residual uterine fluid (histoptroph) is removed using gentle suction, and the lumen is lavaged with sterile lactated ringer’s solution (Figure 4A). Fluid is immediately removed via suction, and 1.0 mL small animal enrofloxacin 2.27% is instilled prophylactically into the uterine lumen before commencing closure. The uterus is sutured with a double layer closure with the mucosal layers closed with 3–0 PDS on a taper needle in a continuous fashion and then oversewn with a Lembert pattern incorporating the muscularis/serosal layers. The stay sutures are then removed, and the uterus is lowered back into coelom (Figure 4B). The body wall is closed via a simple interrupted or simple continuous pattern using 2–0 PDS with a tapered needle to prevent fraying of the peritoneum, the holding layer of the coelom (Figure 4B). The skin is closed with 2–0 PDS on a cutting needle in a horizontal mattress pattern (Figure 4C). This suture pattern is chosen because it is an everting and tension-relieving technique.

**Figure 5:**
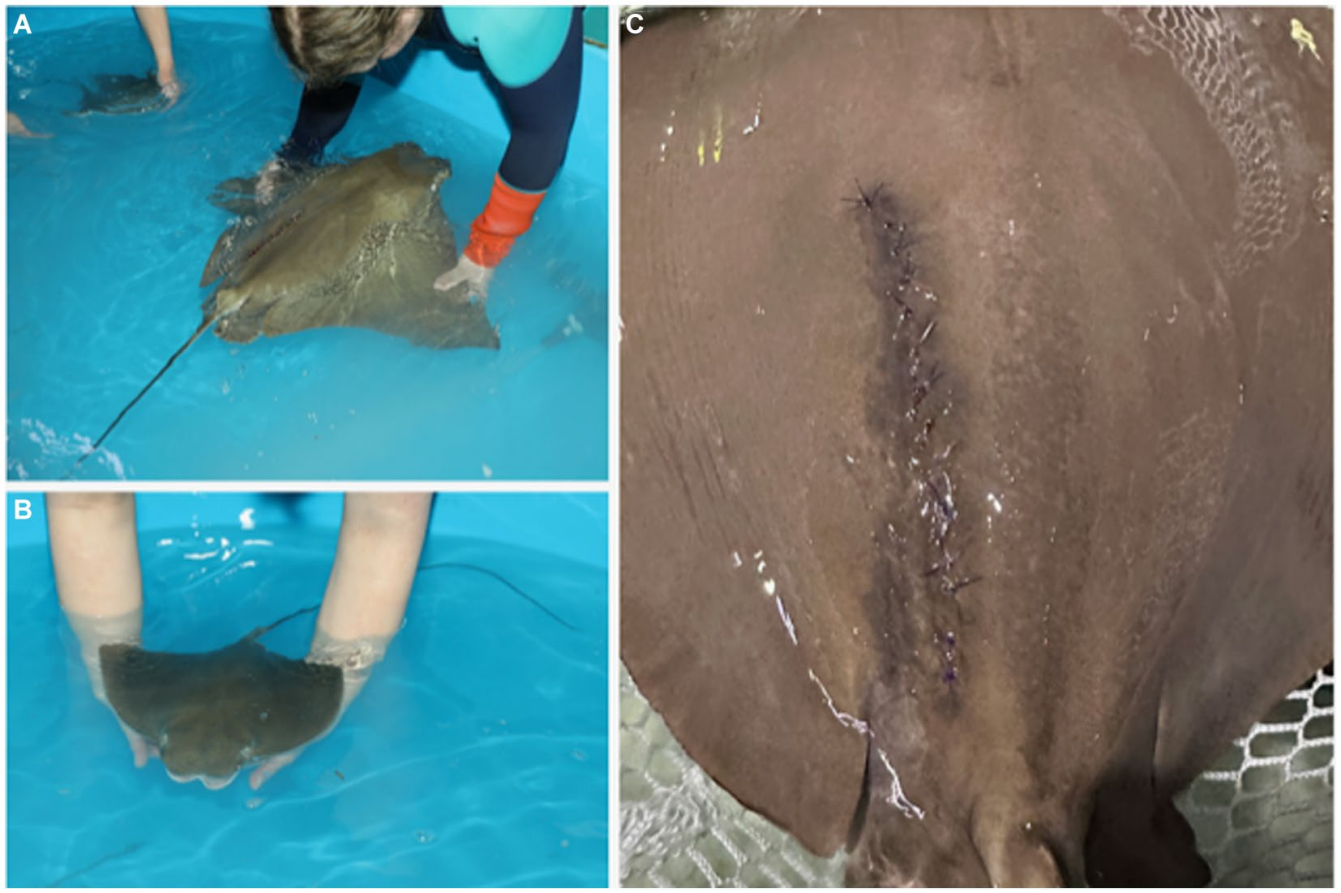
The female **(A)** and neonate **(B)** are transferred to a holding tank with clean, anesthetic-free saltwater for recovery. **(C)** Sutures are removed 4–6 weeks after surgery. The healed incision is characterized by hyperpigmentation that gradually fades.

Rays receive single perioperative intramuscular (IM) injections of meloxicam (0.2 mg/kg), vitamin C (12.5 mg/kg), vitamin B complex (5 mg/kg), and broad-spectrum, long-acting antibiotics [either ceftiofur (6.6 mg/kg) or florfenicol (40 mg/kg)] after closure. The spiracular hoses are removed, and the ray is transferred to an unmedicated holding pool with system seawater for recovery (Figure 5B). Once the ray is responsive to touch, usually 20–30 min, she is returned to exhibit.

The neonate(s) is transferred to a holding tank of anesthetic-free water from the same original source as the female to facilitate recovery (Figure 5A). Supporting the neonate in the water without ventilating and manually and slowly flexing the wings up and down promotes independent swimming (personal observation), which should be observed within 10 min of extraction. The female’s sutures are removed 4–6 weeks after surgery (Figure 5C), and sedation is typically not needed. Removal of sutures earlier than 4 weeks may be possible but was not attempted in this study.

## Results

### Animals

**Figure 6:**
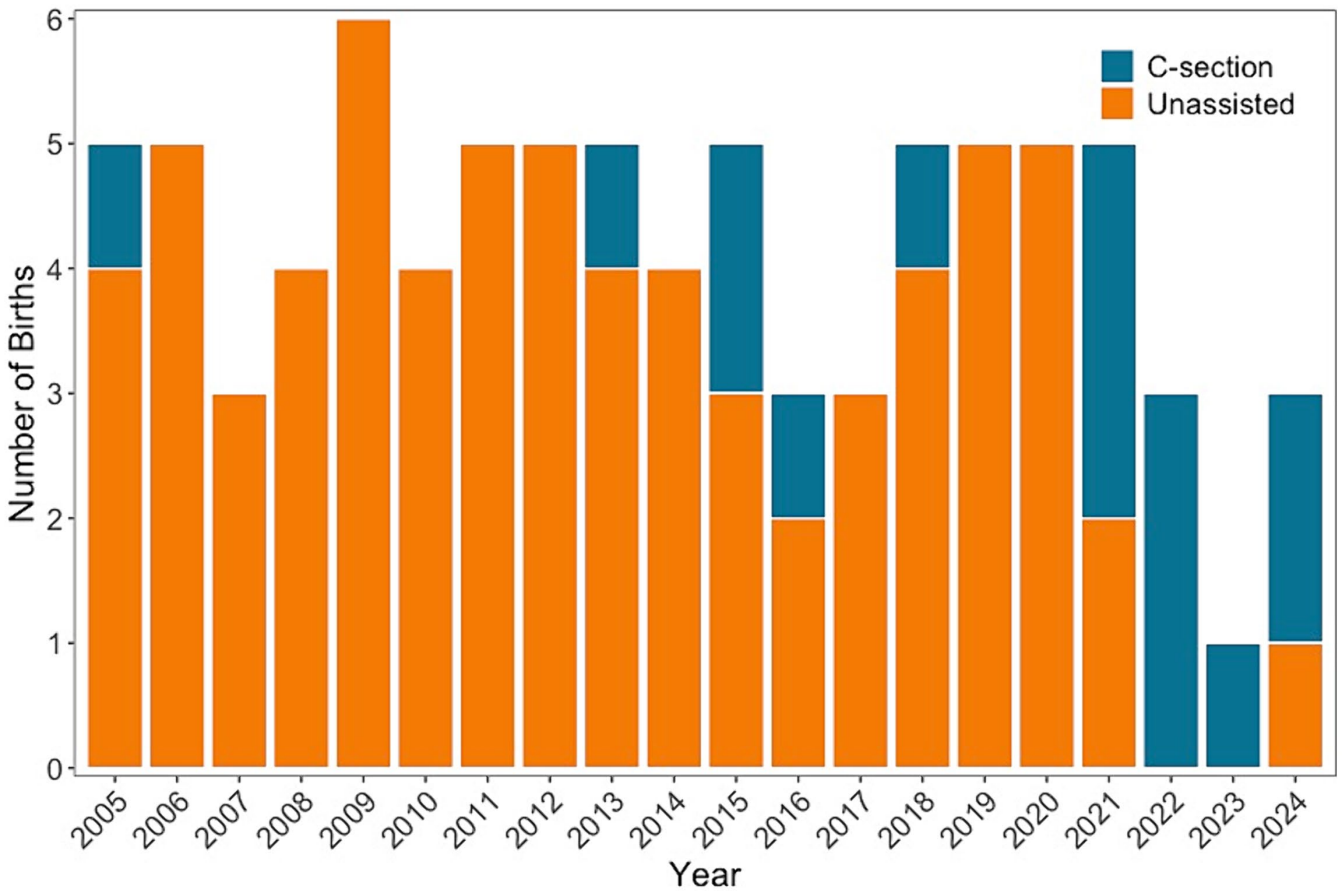
The number of births each year that are unassisted (orange) and C-sections (teal) for seven cownose rays that have had one or more C-sections and are founder rays individuals at Ripley’s Aquarium of the Smokies. Cownose rays are examined monthly to monitor reproductive status, and the number of pregnancies that have required C-sections has increased since 2020.

Fourteen female cownose rays served as founders and have successfully reproduced at Ripley’s Aquarium of the Smokies since it was opened in 2000. In that time, half (*n* = 7) of founder females have undergone one or more C-section procedures. Monthly reproductive monitoring of the cownose ray population has been in place since 2005, and most (60%, 9 of 15) C-sections have occurred in the last 4 years, which represents 20% of the study duration (Figure 6). Among these seven founder rays, there have been 69 parturition events, with 15 (22%) culminating in C-sections. Five of the seven females underwent multiple C-sections: one female had four C-sections, one female had three C-sections, and three females had two C-sections. A brief reproductive history for each female is given in Table 1. The first C-section was performed in 2005; however, detailed medical records for retrospective analysis were not available until 2020, and results are reported accordingly.

**Table 1:** Reproductive history for cownose rays that have had one or more C-sections.

Female	Reproductive history
GB-RB-00-01-F	Unassisted birth in 2006, 2007, 2008, 2009, 2010, 2011, and 2014; C-section in 2015; Unassisted birth in 2017; C-section in 2018; Unassisted birth in 2019, 2020; C-section in 2021
GB-RB-00-04-F	Unassisted birth in 2005, 2007, 2009, 2010, and 2012; C-section in 2013, C-section in 2015; Unassisted birth in 2016, 2018, and 2020; C-section in 2021 and 2023
GB-RB-00-05-F	C-section in 2005; Unassisted birth in 2011 and 2013
GB-RB-00-06-F	Unassisted birth in 2005, 2006, 2008, 2009, 2011, 2012, 2013, 2015, 2018, 2019, and 2020; C-section in 2022 and 2024
GB-RB-00-10-F	Unassisted birth in 2005, 2006, 2008, 2009, 2011, 2012, 2013, 2014, 2015, 2017, 2018, 2019, 2020, and 2021; C-section in 2022 and 2024
GB-RB-00-11-F	Unassisted birth in 2005, 2006, 2009, 2010, 2011, 2012, 2013, and 2014; C-section in 2016; Unassisted birth in 2019; C-section in 2021
GB-RB-00-12-F	Unassisted birth in 2006, 2007, 2008, 2009, 2010, 2012, 2014, 2015, 2016, 2017, 2018, 2019, 2020, and 2021, C-section in 2022; Unassisted birth in 2024

The majority (75%, 9 of 12) of C-sections resulted in live fetuses. The clinical presentation of females that underwent C-sections resulting in live fetuses included partial or marked pelvic fin edema and cloacal prolapse (78%, 7 of 9), which were consistently observed in concert. The urogenital papilla was noted to be prominent for one of the seven females with marked edema and prolapse. She was also observed to be increasingly lying on the bottom of the enclosure in the days before her C-section, highlighting a behavior change presumed to be related to her reproductive condition. The duration of stage 5, late pregnancy, was 3 months or more for for 6 C-sections including 5 females suggesting over gestation and 2 of these females developed a pressure ulcer on their ventral coelom. In addition to maternal concerns as a reason for C-section, the uterine fluid transitioned from a normal hypoechoic and homogeneous appearance on ultrasound to a very hyperechoic and heterogeneous appearance for two females and pregnancies (22%, 2 of 9), which was a health concern for the fetus that prompted C-section.

Neonates usually swam under their own power shortly after birth. They were transferred either to a nursery enclosure or a holding pool with other neonate rays to isolate them from large rays in the exhibit, thereby preventing injury and predation. Rays delivered naturally or by C-section commenced feeding on their own approximately 5–7 days after birth (personal observation). After the initial period of inappetence, they ate readily using general broadcast feeding methods. Cownose ray neonates were individually identified with passive integrated transponders (PIT) when 3–4 months old. At this age, they were large enough to be released into the exhibit with other fish without risk of predation.

### Complications

No mortalities were associated with C-section procedures, but surgical site complications were observed for two of nine procedures involving two out of seven females. Both complications, one minor and the other major, were dehiscence. The minor complication was partial dehiscence near the center of the C-section incision and was observed 5 days post-C-section. On day seven postprocedure, the body wall was still intact, and malachite green was applied to the central open area. Weekly rechecks confirmed healing progression, and no further treatment was required.

The major complication was provoked by tankmates and occurred for a ray that was returned post-C-section to its habitat, which included other rays and porkfish (*Anisotremus virginicus*). After 5 days, the porkfish aggravated the surgical site by chewing out the skin sutures. The porkfish were immediately removed to a different tank to prevent further irritation of the surgical wound. The size of the ray’s defect prevented reclosing the skin, and the lesion was irrigated with copious amounts of iodine and malachite green. After 2 days, a complete wound dehiscence occurred. The ray was anesthetized, and the wound was irrigated and prepped with iodine. The remaining body wall sutures were removed, and the edge was debrided. The body wall was closed with 2–0 PDS in a simple interrupted pattern. The cranial 2/3 of the skin was successfully reapposed with 2–0 PDS in a horizontal mattress pattern. The caudal 1/3 was partially closed with 2–0 PDS in a horizontal mattress pattern. Ceragyn® Wound and Skin spray (Purishield Life Sciences, LLC, CA, United States) was applied, followed by malachite green, Cavilon^™^ No Sting Barrier Film (3 M, Medical Solutions Division, MN, United States), and finally, liquid bandage. Perioperative IM injections of vitamin C (12.5 mg/kg) and ceftiofur (6.6 mg/kg) were given. Beginning 5 days after the reclosure, malachite green was applied, and enrofloxacin (10 mg/kg) was dosed daily for 5 days. The wound was rechecked on day 7 after reclosure and showed continued improvement. Malachite green and enrofloxacin (10 mg/kg) were dosed again daily for 5 days. The wound was rechecked on day 14 after reclosure and showed continued improvement. Malachite green, enrofloxacin (10 mg/kg), and vitamin C (12 mg/kg) were dosed daily for 5 days. On day 21 postreclosure, there was significant skin regeneration over the wound bed, and a final course of enrofloxacin (10 mg/kg) was continued daily for 5 days. The sutures were removed 28 days after reclosure.

In the long term, none of the rays exhibited complications related to C-sections. One female (GB-RB-00-11) died a year after her procedure of an unrelated cause. Rays were not isolated after C-section and instead returned to their exhibit. They resumed normal swimming patterns, activity, and appetite the same day as their C-section procedure. The surgical site entirely healed within 4–6 weeks, but the incision was still visible as a hyperpigmented scar for at least a year post-surgery.

### Outcomes

Females (*n* = 7) were in managed care for an average [±standard deviation (SD)] of 15.4 ± 6.3 years before their first C-section was performed. Five of seven females who had C-sections were able to deliver normally and unassisted in future pregnancies. Two females (GB-RB-00-06 and GB-RB-00-12) had a long history of unassisted births beginning in 2005 and 2006, and for both, only their most recent two pregnancies (2022 and 2024) required assistance via C-section. Most births (78%, 53 of 69) occurred before a female’s first C-section. The average number of unassisted births prior to C-section was 8 ± 4 (range 0–14), and the average number of unassisted births after a female’s first C-section was 1 ± 1 (range 1–3).

No mortalities have occurred among pregnant females because of dystocia, but 25% (3 of 12) of procedures were conducted secondary to fetal death. Death was confirmed using ultrasonography, characterized by lack of fetal heartbeat, lack of movement of gill arches indicative of respiration, absence of fetal movement, turbid uterine fluid, and a uterine mucosa with trophonema of variable lengths and thickness. No complications were noted in the short-term period for delivered neonates. One male newborn was remarked to be large and had difficulty unfolding his wings after extraction. Passive range of motion exercises were completed thrice daily to increase his range of motion.

Because cownose rays reproduce readily, there was almost always a group of neonates isolated within the main exhibit or in a holding tank of the exhibit. The isolation was for protection from other adult rays that share the exhibit, and it also facilitates monitoring feeding. The long-term outcome of C-section neonates was hampered by the delay in individual identification using PIT tags except for a 2015 C-section where the neonate is known unequivocally and alive at the time of publication, nearly 10 years post-procedure.

## Discussion

Reproduction in managed care is a requirement and responsibility for achieving self-sustaining populations and supporting conservation and education initiatives. Not all species reproduce readily in aquariums, but for those that do, a thorough understanding of the reproductive anatomy and cycle is paramount to developing procedures to circumvent complications arising from reproductive efforts. For pregnant cownose rays suffering from over-gestation, dystocia, or fetal death, a C-section, as described in this report, is a lifesaving procedure. Most females that underwent C-sections had a history of successful reproduction with unassisted births both before and after their C-section procedure(s). Females that underwent C-sections were already mature and had been in managed care for an average of more than 15 years before their first C-section. Two females had delivered unassisted since 2005, but their two most recent pregnancies required C-sections. The monitoring efforts of female cownose rays at Ripley’s Aquarium of the Smokies have not changed since 2005, but C-sections have increased in frequency during the last 4 years. This suggests that complications with gestation may be more prevalent as rays age, which may be an important consideration for the reproductive management of older rays. However, dependency on assisted birth is not necessarily a long-term problem because females are able to deliver naturally after C-section.

Determining when to intervene and assist with delivery is important because waiting too long may result in the loss of the neonate and potentially the female. Ideally, this decision is data-driven and based on knowledge of the female’s reproductive history and status. The cownose rays at Ripley’s Aquarium of the Smokies breed readily, and their reproductive status has been monitored monthly via ultrasonography since 2005. Females are assigned a reproductive stage (stages 0–5) based on the contents and appearance of the uterus (6). In the first cases of dystocia observed (2005 and 2013), a C-section was performed retroactively to remove stillborn young. Later, C-sections were performed proactively, largely due to monthly reproductive monitoring of the population, resulting in live young being extracted. For C-sections to be used proactively, a commitment to routine reproductive and health monitoring is needed to understand when gestation began and accurately predict the delivery date, which can then be used to help decide when intervention is warranted.

Adapting a procedure for use with a new species requires flexibility, attention to detail, and a willingness to recognize the pitfalls of a procedure and document how to avoid them. Customized equipment and lessons learned from procedure complications enabled the refinement of the surgical technique and recovery requirements to ensure favorable outcomes for future procedures. A portable procedure table was built for stingray medical procedures that included a surgery area and recirculating water to maintain anesthesia until the end of the procedure by delivering water to both spiracles with the rate of delivery infinitely adjustable using valves in the water lines. The surgical drape was a non-permeable, sterilized plastic barrier instead of a standard paper surgical drape. Keeping recovering rays with sutures isolated from fish that may irritate or disrupt the healing suture line was essential to prevent infection and enlargement of the wound.

This report details the procedure and long-term outcomes of C-sections for cownose ray females. This demonstrates that the procedure does not impact the rays’ future reproduction. C-sections were increasingly required as females aged, which is important in monitoring gestation length and clinical signs of dystocia and may be a consideration for managing mixed populations in aquariums.

## Data Availability

The original contributions presented in the study are included in the article/supplementary material, further inquiries can be directed to the corresponding authors.
